# Water kefir as a promising low-sugar probiotic fermented beverage

**DOI:** 10.1186/2049-3258-72-S1-P1

**Published:** 2014-06-06

**Authors:** David Laureys, Luc De Vuyst

**Affiliations:** 1 Research Group of Industrial Microbiology and Food Biotechnology, Faculty of Sciences and Bioengineering Sciences, Vrije Universiteit Brussel, B-1050 Brussels, Belgium

## Background

Water kefir is a slightly sweet, acidic, fruity, sparkling, and slightly alcoholic fermented beverage produced with water kefir grains, the latter consisting of polysaccharides and micro-organisms. The micro-organisms involved in water kefir fermentation comprise yeast, lactic acid bacteria, bifidobacteria, and acetic acid bacteria. Some strains of micro-organisms in water kefir might possess probiotic activity. Also, water kefir is a beverage with relatively low sugar content, providing an interesting alternative to sugary soft drinks. To be able to exploit water kefir for its probiotic potential or as a low-sugar soft-drink, in-depth research is needed to unravel the species diversity and community dynamics of water kefir fermentation. In particular, substrate consumption and metabolite production in water kefir have not been studied until now.

## Materials and methods

Water kefir fermentation processes were followed as a function of time for 192 h. At each sampling point, the pH, microbial counts (lactic acid bacteria, yeast, and acetic acid bacteria), and residual substrate and metabolite concentrations were measured, including volatile aroma compounds. The species diversity was unravelled through both culture-dependent and culture-independent techniques.

## Results

Species diversity analyses indicated that the most important microbial species were *Lactobacillus casei/paracasei*, *Lactobacillus harbinensis*, *Lactobacillus hilgardii*, *Bifidobacterium psychraerophilum*/*crudilactis*, *Saccharomyces cerevisiae*, and *Dekkera bruxellensis*. This microbial species diversity was similar in the water kefir liquor and on the water kefir grains, and remained stable during the whole fermentation process. Some strains of these species, such as *Lb. casei* and *Bifidobacterium* spp., might possess probiotic activities. Sucrose, the major substrate of the fermentation was completely converted after 24 h of fermentation, which coincided with the production of the water kefir grain polysaccharide. The main metabolites of the fermentation were ethanol and lactic acid, whereas glycerol, acetic acid, and mannitol were produced in low concentrations. The most prevailing volatile aroma compounds (relative to their threshold values) were ethyl acetate, isoamyl acetate, ethyl hexanoate, ethyl octanoate, and ethyl decanoate. These are fruity esters which might have a positive impact on the aroma of the end-product.

## Conclusions

In this study the species diversity, community dynamics, substrate consumption, and metabolite production during water kefir fermentation were described in detail. This work provides a basis for further developments of water kefir as healthy, low-sugar, probiotic fermented beverage.

